# NeuraGraph: a visual workflow framework for reproducible biomedical text mining with LLM-agents

**DOI:** 10.1093/bioadv/vbag240

**Published:** 2026-08-24

**Authors:** Lei Zhao, Changning Ren, Xinning Liu, Li Han, Yingnan Fan, Ling Kang, Quan Guo

**Affiliations:** Neusoft Research Institution, Dalian Neusoft University of Information, Dalian, 116023, China; Software Department, Dalian Neusoft University of Information, Dalian, 116023, China; Software Department, Dalian Neusoft University of Information, Dalian, 116023, China; Software Department, Dalian Neusoft University of Information, Dalian, 116023, China; Software Department, Dalian Neusoft University of Information, Dalian, 116023, China; Neusoft Research Institution, Dalian Neusoft University of Information, Dalian, 116023, China; Neusoft Research Institution, Dalian Neusoft University of Information, Dalian, 116023, China

## Abstract

**Summary:**

Biomedical text mining increasingly demands hybrid pipelines integrating traditional NLP toolkits and LLM-based agents, yet building reproducible and modular workflows remains challenging. We present NeuraGraph, a lightweight open-source Python framework for reproducible biomedical text mining workflows integrating traditional NLP components and LLM-based agents. NeuraGraph provides hierarchical workflow composition through reusable subgraphs, built-in batch experimentation with standardized evaluation, and an LLM-powered reporting and refinement engine for workflow optimization. We evaluated NeuraGraph on named entity recognition and chemical-induced disease relation extraction using the BioCreative V CDR benchmark. An optimized relation extraction workflow improved Micro-F1 from 0.656 to 0.673 and Macro-F1 from 0.642 to 0.666 through reporting-guided refinement. Compared with functionally equivalent custom implementations, NeuraGraph reduced implementation effort from 366 to 135 source code lines for relation extraction workflows while maintaining comparable extraction performance. The framework facilitates systematic comparison, reproducible evaluation, and rapid development of hybrid biomedical text mining pipelines.

**Availability and implementation:**

NeuraGraph is implemented in Python 3.12+ and released under the MIT license, with all dependencies listed in a requirements.txt file for straightforward installation. Source code and documentation are available at https://github.com/tyrone1979/neuragraph. The software runs natively on Linux, macOS, and Windows.

## 1 Introduction

Biomedical text mining is a cornerstone of translational bioinformatics, the discipline that bridges basic biomedical research and clinical practice (Cohen and Hunter 2013). It enables the extraction of actionable insights, e.g. chemical-disease relations, gene-phenotype associations from unstructured scientific literature (Lan *et al.* 2025, Xu and Sankar 2025). Text mining has been successfully applied across the translational spectrum: in drug discovery (Öztürk *et al.* 2018), pharmacovigilance (Karimi *et al.* 2015), and precision medicine from clinical narratives (Wei *et al.* 2020).

Modern mining pipelines increasingly adopt a hybrid paradigm that combines the reproducibility of traditional modular NLP tools such as Flair (Akbik *et al.* 2019) with large language model based agents (Zhao *et al.* 2025). Recent surveys have documented this trend, showing that hybrid approaches consistently outperform purely traditional or purely LLM-based systems (Huang *et al.* 2025). Scenario-based prompt design has further demonstrated the effectiveness of LLM-based reasoning for document-level biomedical relation extraction (Zhao *et al.* 2025). This integration unlocks state-of-the-art performance for tasks such as named entity recognition (NER) and relation extraction (RE) on biomedical benchmarks (Jin *et al.* 2025, Lv *et al.* 2025), but introduces critical engineering challenges for biomedical researchers without extensive coding expertise.

A central bottleneck in hybrid pipeline development is the orchestration of heterogeneous components into reproducible, evaluable workflows. General purpose workflow frameworks like LangChain and LangGraph (LangChain Team 2024) provide rich orchestration primitives but demand substantial coding effort, offer no built‑in support for biomedical benchmarks, and lack automated experiment logging and evaluation. On the other hand, low‑code platforms such as Dify (Dify 2025) are designed for production deployment, introduce infrastructure dependencies such as Docker, and do not facilitate fine‑grained, per‑instance error analysis required for methodical research. As a result, practitioners rely on ad-hoc scripting, hindering reproducibility and systematic comparison of extraction strategies.

To address these gaps, we present NeuraGraph, a lightweight, open-source workflow framework designed for reproducible biomedical text mining with hybrid LLM-agent pipelines. Building on a LangGraph core, NeuraGraph introduces four integrated capabilities for domain researchers: (i) formal input validation to ensure workflow graph correctness; (ii) recursive subgraph composition for hierarchical pipeline design and reuse; (iii) batch experimentation with standardized evaluation metrics and persistent execution artifacts; (iv) an LLM-powered reporting and refinement engine for diagnostic analysis and iterative prompt-level optimization. These capabilities enable systematic construction, execution, and comparison of biomedical text mining workflows under consistent experimental conditions.

NeuraGraph is engineered for immediate usability. It ships with rich collection of built-in agents, reusable subgraphs, reference workflows, callable tools, and native parsers for established biomedical benchmarks such as BioCreative V CDR corpus (Li *et al.* 2016) and the ChemDisGene (Zhang *et al.* 2022). A browser-based visual editor enables rapid workflow assembly; a secure plugin system supports one-line integration of local biomedical models (e.g. Flair/HunFlair2); and every batch run persists workflow configuration, execution traces, predictions, and metrics as version-controllable JSON artifacts. An experiment wizard supports separate test and tuning datasets and optimization guided by tuning-set reports, without manual orchestration coding. Unlike general orchestration and deployment-oriented platforms, NeuraGraph combines formal workflow validation, recursive subgraph reuse, integrated evaluation, and tuning-driven refinement in a lightweight pure-Python environment, reducing engineering overhead while supporting full reproducibility.

NeuraGraph does not aim to introduce a novel biomedical extraction model. Instead, its primary contribution is a reproducible low-code workflow framework that enables biomedical researchers to rapidly assemble, evaluate, reproduce, and refine hybrid LLM-agent extraction pipelines under standardized experimental conditions.

## 2 Implementation

NeuraGraph is implemented in Python 3.12+ and distributed under the MIT open-source license, with dependencies listed in a requirements.txt. The core framework runs on standard research hardware (e.g. laptops with Intel i7 and 16 GB RAM) with no mandatory Docker dependencies. LLM inference is optional and decoupled from core execution, accessed via lightweight connectors to local GPU servers (e.g. Ollama) or cloud services through OpenAI-compatible endpoints.

The framework provides integrated modules for end-to-end biomedical text mining pipeline development and validation:

1. **Input and output.** NeuraGraph accepts end-to-end biomedical text mining by accepting raw text or annotated corpora (e.g. CDR, ChemDisGene) together with workflow graphs defined in a visual editor or JSON format. Users may optionally edit LLM prompt templates via the visual interface, while default prompts are provided. Outputs include per-instance predictions, evaluation metrics (micro/macro precision, recall, F1), and a self-contained JSON artifact recording workflow configurations, execution traces, intermediate outputs, and results. For unlabeled data, the same artifact structure is preserved without metrics.
2. **Built-in components.** The distribution includes 67 pre-built agents (36 LLM-based, 31 programmatic/PGM), 10 reusable subgraphs, 23 workflows, 12 tools, and native parsers for CDR, ChemDisGene. The complete inventory is documented in Supplementary Section 4.
3. **Recursive subgraph composition.** Complete workflows can be reused as subgraphs for hierarchical design, e.g. sentence-level NER within document-level relation extraction. Beyond linear execution, NeuraGraph supports conditional branching (if/else-style routing) and graph-level routing based on intermediate outputs, enabling workflows to dynamically adapt execution paths (e.g. filtering entities before relation extraction or selectively invoking subgraphs for specific input patterns). All intermediate outputs are stored as structured JSON artifacts to ensure reproducibility.
4. **Plugin integration and sandbox execution.** Local biomedical models (e.g. Flair) are integrated via a lightweight plugin interface built on a unified Plugin base class. Heavy-weight plugins run in isolated sandbox sidecar processes with dedicated virtual environments and communicate via a restricted HTTP interface, ensuring dependency isolation and failure containment. Each plugin typically requires ≤50 lines of code.
5. **Batch experiments.** NeuraGraph supports batch execution with version-controlled workflows. Each run produces a persistent JSON artifact for full reproducibility.
6. **LLM-powered reporting and refinement engine.** Batch runs are analyzed to generate Markdown reports with metrics and failure patterns. A tuning-and-test workflow uses diagnostic feedback to guide prompt-level optimization, followed by re-evaluation on a held-out test split with baseline comparison.

**Figure 1:**
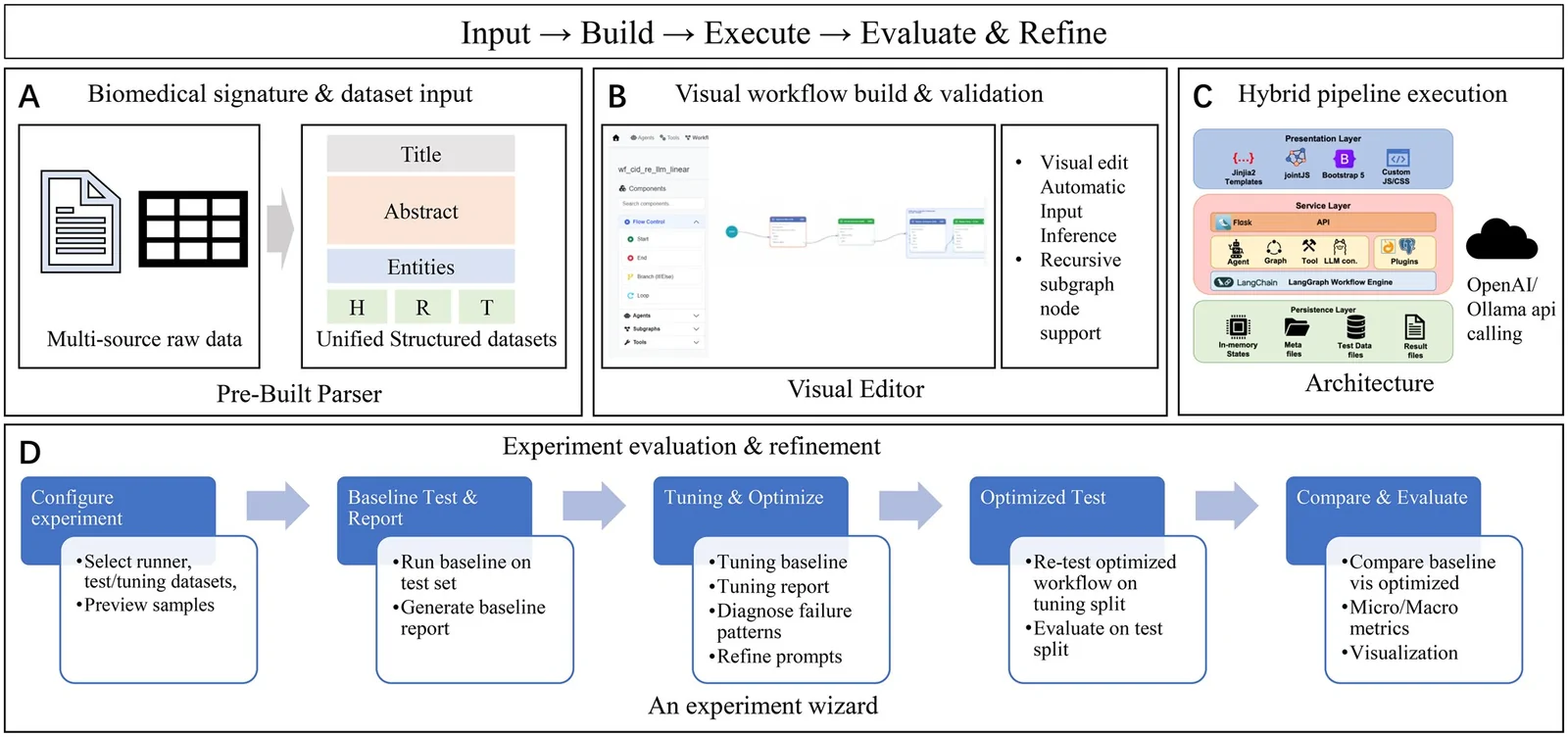
Overview of the NeuraGraph framework for reproducible biomedical text mining. (A) Biomedical text and benchmark datasets (e.g. CDR and ChemDisGene) are imported through native parsers and converted into structured inputs. (B) Workflows are assembled and validated in a visual editor with automated input inference and recursive subgraph composition. (C) Hybrid workflow execution integrates programmatic components, biomedical NLP models, and LLM-based agents through local execution and remote LLM inference services (e.g. Ollama or OpenAI-compatible APIs). (D) An LLM-powered reporting and refinement engine supports iterative workflow optimization through a tuning-and-test workflow, including workflow configuration, baseline evaluation, diagnostic report generation, prompt-level refinement, optimized workflow execution, and comparative performance assessment using micro- and macro-averaged precision, recall, and F1 metrics.

An overview of the NeuraGraph framework is shown in Fig. 1. Additional implementation details, workflow examples, plugin development guidelines, and complete component inventories are provided in the Supplementary Material.

Representative workflow examples are provided in the Supplementary Material, including a named entity recognition (NER) workflow (Figure S4), a chemical-induced disease (CID) relation extraction workflow (Figure S5), and an end-to-end workflow combining Flair-based NER with optimized CID relation extraction (Figure S6).

## 3 Results

### 3.1 Biomedical text mining workflow evaluation

NeuraGraph is evaluated on chemical/disease named entity recognition (NER) and chemical-induced disease (CID) relation extraction (RE) tasks using the BioCreative V CDR benchmark (Li *et al.* 2016).

All workflows used identical core components, including HunFlair2 (Akbik *et al.* 2019) for NER, GPT-4o/Mistral 7B for relation verification, and micro/macro precision, recall, and F1-score for evaluation.

To demonstrate practical applicability in a realistic pharmacological text mining scenario, an end-to-end workflow was constructed for automatic extraction of chemical-induced disease associations from biomedical literature. Biomedical abstracts are first processed using a sentence-level NER subgraph to identify chemical and disease entities. Candidate chemical–disease pairs are then generated programmatically and verified using LLM-based relation extraction agents. The relation verification prompt follows the prompting strategy proposed in our previous biomedical relation extraction study (Zhao *et al.* 2025), and is implemented as a configurable workflow component within NeuraGraph.

For comparison, a second end-to-end workflow replaced the NER component with PubTator3 (Wei *et al.* 2024) while retaining the same downstream relation extraction workflow. This design enabled direct assessment of how different entity recognition components affect end-to-end CID extraction performance.

Recursive subgraph composition enabled reusable sentence-level and pairwise processing components while simplifying workflow orchestration and reducing post-processing complexity.

### 3.2 Quantitative performance and workflow efficiency

Table 1 summarizes the quantitative performance on NER and RE, and end-to-end CID extraction.

**Table 1:** Quantitative performance evaluation on CDR benchmark.

Method	Micro-P	Micro-R	Micro-F1	Macro-P	Macro-R	Macro-F1
Flair NER	0.725	0.840	0.779	0.737	0.844	0.779
LLM NER	0.640	0.673	0.656	0.672	0.690	0.667
LLM RE (baseline)	0.583	0.750	0.656	0.610	0.757	0.642
LLM RE (optimized)	0.603	0.761	0.673	0.626	0.791	0.666
E2E (Flair NER to optimized RE)	0.543	0.765	0.635	0.632	0.781	0.663
E2E (PubTator3)	0.281	0.660	0.394	0.344	0.699	0.422

Notes: All metrics are computed at the document-level aggregation, followed by cross-document micro/macro averaging.

For NER, the Flair-based pipeline achieves the best performance (Micro-F1 = 0.779), while the LLM based pipeline is lower (Micro-F1 = 0.656), indicating that domain-specific models remain advantageous for entity extraction.

For CID relation extraction, the baseline LLM pipeline achieves Micro-F1 = 0.656. After reporting-guided refinement, performance improves to 0.673, showing consistent gains in both precision and recall.

For end-to-end evaluation, combining Flair NER with optimized RE yields Micro-F1 = 0.635. In comparison, the PubTator3-based pipeline achieves lower performance (Micro-F1 = 0.394), highlighting the impact of NER quality on downstream RE performance.

Implementation effort was additionally quantified using source code line counts under functionally equivalent settings (Table 2).

**Table 2:** Implementation effort comparison.

Task	Implementation Effort (source code lines)		
	NeuraGraph	Custom Python code	Dify
Chemical/Disease NER	35	361	101
Chemical-induced disease RE	135	366	232

Notes: (i) All three implementations achieve functionally equivalent results on the CDR benchmark under the same experimental setting, including identical input/output formats, shared evaluation metrics (micro/macro precision, recall, and F1-score), and consistent use of core models (Flair-HunFlair2 for NER and Mistral-7B/GPT-4o via remote Ollama for LLM-based reasoning). (ii) The primary difference lies in implementation approach: NeuraGraph uses visual assembly and built-in components; custom Python requires manual coding of orchestration, logging, and metric aggregation; Dify demands explicit prompt configuration, API wiring, and Docker setup for local models. (iii) Code lines exclude blank lines and comments.

NeuraGraph significantly reduces engineering effort compared to custom implementations. For CID relation extraction, NeuraGraph requires 135 lines versus 366 lines in custom Python, while maintaining identical input/output formats and evaluation metrics.

These results demonstrate that NeuraGraph reduces engineering overhead while preserving functional equivalence across biomedical text mining pipelines.

### 3.3 Recursive subgraph benefits: a concrete example

To illustrate recursive subgraph composition, we use the CID relation extraction workflow as an example.

Candidate chemical–disease pairs are processed through a reusable relation-verification subgraph that encapsulates reasoning and normalization steps.

Compared with flat pipelines, where verification and post-processing logic are repeatedly embedded, the subgraph isolates these operations into a reusable execution unit, improving modularity and reducing redundancy.

The same subgraph is applied to all candidate pairs, ensuring consistent execution and standardized outputs. It automatically converts predictions into structured relations and aggregates document-level results, eliminating manual post-processing.

This design avoids inconsistencies introduced by repeatedly implementing normalization and filtering logic across iterations, and by ensuring that each invocation operates on an isolated, stateless execution context with fixed input/output contracts. As a result, intermediate representations do not accumulate or diverge across loop executions, which improves reproducibility and reduces variability in downstream aggregation.

The complete workflow is shown in Figure S5, while the end-to-end NER-to-RE workflow is shown in Figure S6.

### 3.4 LLM-powered reporting, refinement, and reproducibility

The LLM-powered reporting and refinement engine analyzes persisted workflow artifacts to identify performance bottlenecks and generate structured optimization suggestions. For each experiment, NeuraGraph stores workflow configurations, execution traces, intermediate outputs, predictions, and evaluation metrics in a self-contained JSON artifact.

These artifacts are processed to generate Markdown reports containing quantitative summaries, error distributions, and failure-pattern descriptions.

In the CID workflow, the reporting engine highlights false-positive disease associations and ambiguous entity normalization as key sources of precision loss. It identifies cases where chemical–disease co-occurrence is incorrectly interpreted as causal evidence, leading to over-predicted relations. These observations align with qualitative inspection of representative errors, indicating that the generated reports provide interpretable diagnostic signals for workflow refinement.

Based on these signals, reporting-guided refinement process proposes prompt-level modifications for the relation verification agent and generates optimized workflow variants for re-evaluation.

Using the built-in tuning-and-test workflow, the optimized configuration improves Micro-F1 from 0.656 to 0.673 and Macro-F1 from 0.642 to 0.666 (Table 1). This demonstrates that report-guided refinement can improve relation verification performance without modifying the underlying workflow structure.

For end-to-end evaluation, the optimized RE pipeline combined with Flair-based NER achieves Micro-F1 = 0.635 and Macro-F1 = 0.663. In comparison, the PubTator3-based pipeline yields lower performance (Micro-F1 = 0.394, Macro-F1 = 0.422), highlighting the impact of entity extraction quality on downstream relation extraction.

All executions generate self-contained JSON artifacts, enabling reproducible re-execution under identical environments.

### 3.5 Generalizability

NeuraGraph is designed as a general-purpose biomedical text mining framework beyond the CDR benchmark, supporting the ChemDisGene corpus through dedicated parsers, task-specific agents, and complete evaluation workflows. The framework covers a wide range of biomedical information extraction tasks, including chemical–gene relations, gene–disease associations, named entity recognition, entity normalization, synonym and hypernym detection, dependency-based extraction, and biomedical knowledge graph construction.

Generalizability is enabled by a modular architecture. Dataset ingestion is handled via pluggable parsers, while workflow components operate on standardized data schemas. New datasets can be incorporated by implementing a dataset-specific parser without modifying the execution engine.

External biomedical models can be integrated through the plugin system, enabling reuse of existing agents, subgraphs, and evaluation modules under unified execution and reporting standards.

End-to-end examples for CDR and ChemDisGene workflows, together with JSON artifacts and evaluation reports, are provided in the Supplementary Material. These demonstrate consistent execution and evaluation across datasets.

Overall, NeuraGraph functions as a reproducible experimental platform for biomedical text mining, supporting standardized evaluation, structured error analysis, and systematic comparison of hybrid NLP–LLM pipelines.

## 4 Limitations

Despite its demonstrated benefits for reproducible biomedical text mining, NeuraGraph has several limitations that should be acknowledged.

**Single-machine scalability.** The framework is optimized for consumer-grade hardware and does not yet support distributed execution.

**Limited empirical scope.** Evaluation focuses on the CDR benchmark. Broader validation on clinical and large-scale biomedical datasets remains future work.

**Data format constraints.** Native support is provided for CDR, ChemDisGene, and plain text. New datasets require lightweight custom parsers.

Future work will extend distributed execution, expand dataset coverage, and enhance large-scale evaluation.

## 5 Conclusion

NeuraGraph establishes a lightweight workflow framework for reproducible biomedical text mining with hybrid NLP and LLM-agent pipelines. It integrates workflow validation, recursive subgraph composition, standardized evaluation, and an LLM-powered reporting and refinement engine into a unified Python environment.

Experimental results on the BioCreative V CDR benchmark show that NeuraGraph preserves model performance while enabling reporting-guided optimization that improves relation extraction. The framework reduces engineering overhead and supports reproducible evaluation of hybrid pipelines.

As an open-source platform for standard research hardware, NeuraGraph lowers the barrier for constructing and evaluating biomedical text mining workflows and facilitates systematic comparison of extraction strategies.

## Supplementary Material

vbag240_Supplementary_Data

## Data Availability

Source code and documentation are available at https://github.com/tyrone1979/neuragraph.
