# Supplementary material for: NeuraGraph: a visual workflow framework for reproducible biomedical text mining with LLM-agents
Source: Bioinform Adv. 2026 Aug 24;6(1):vbag240. doi: 10.1093/bioadv/vbag240 (PMC13557776; doi:10.1093/bioadv/vbag240)
Supplement: Supplementary file 1 [file vbag240_supplementary_data.docx]

# **Supplementary Material**

## 1. Software architecture

NeuraGraph adopts a three‑layer architecture, as illustrated in **Figure S1**. The **Presentation Layer** provides a browser-based visual editor for drag-and-drop workflow assembly with real-time input-inference validation. The **Service Layer** consists of the Flask API, LangGraph orchestration engine, plugin system, and LLM connectors. It is responsible for executing batch workflows, managing experiment configuration, supporting the experiment wizard (including tuning-set and test-set execution, reporting, and reporting-guided refinement), and optionally deploying sandbox sidecar processes for compute-intensive local models. The **Persistence Layer** manages all persistent artifacts, including versioned workflow and agent definitions stored under meta/, per-experiment execution states stored in states.json, and automatically generated Markdown reports stored under result/.

**Figure S1.** High-level architecture of NeuraGraph, illustrating the three-layer design: (A) Presentation Layer, providing a browser-based visual editor for workflow construction and validation; (B) Service Layer, including Flask API, LangGraph orchestration, plugin system, LLM connectors, and experiment execution engine supporting batch workflows and reporting-guided refinement; and (C) Persistence Layer, responsible for storing versioned workflow definitions, execution states, and generated experiment reports.

## 2. Core Features Implementation

**Feature 1 Automatic input inference (Algorithm S1).** Given a directed workflow graph *G*= (*V*, *E*) where each node declares its inputs and outputs, NeuraGraph computes the minimal set of external inputs required to invoke *G*. This ensures that all data dependencies are satisfied prior to execution and prevents runtime errors caused by missing upstream inputs.

| **Algorithm S1: Automatic input inference** | |
| --- | --- |
| **Input:** graph identifier *g*_id_ | |
| **Output:** A sorted list of global input variable names | |
| **Steps:** | |
| 1 | Load the graph metadata $M\left( g_{id} \right)$to obtain the list of node list $\left[ v_{1},\ldots,v_{n} \right]$. |
| 2 | Initialize three sets: $O_{all}\leftarrow\emptyset$, $I_{non-first}\leftarrow\emptyset$, $I_{first}\leftarrow\emptyset$ |
| 3 | For each node $v_{i}$ (index $i$ from $0$to $n-1$):  a. Load the agent metadata $A_{i}\leftarrow M\left( v_{i} \right)$ .  b. If $A_{i}$ defines an output field $o$, add $o$ to $O_{all}$.  c. If $i$= 0, add all inputs of $A_{i}$ to $I_{first}$; otherwise, add all its inputs of $A_{i}$ to $I_{non-first}$. |
| 4 | Compute the uncovered inputs of non‑first nodes:  $I_{uncovered}\leftarrow I_{non-first}\backslash O_{all}$ . |
| 5 | The global input set is: $I_{global}\leftarrow I_{first}\cup I_{uncovered}$. |
| 6 | Return sort ($I_{global}$). |

**Feature 2 Recursive subgraph composition (Algorithm S2).** NeuraGraph supports hierarchical workflow construction through loop-type flow nodes, each of which references a reusable subgraph (e.g., sentence-level named entity recognition or pairwise relation verification subgraphs). For each element in a shared execution state (e.g., sentences, candidate pairs), the loop node executes the referenced subgraph once and merges list- or dictionary-type outputs into the parent workflow state after completion.

| **Algorithm S2: Recursive Subgraph Invocation(_call_agent)** | |
| --- | --- |
| **Input:** Agent identifier $a$; execution state $\sigma\in\Sigma$ | |
| **Output:** a partial state update $\Delta\sigma$ | |
| **Steps:** | |
| 1 | Load the Agent metadata $A\leftarrow M\left( a \right)$ |
| 2 | Type Check: if $A.type \neq“subgraph”$: Return $invoke(A,\sigma)$ |
| 3 | **Subgraph Handling (**$A.type =“subgraph”$**):**  a. Subgraph Load: $G_{a}\leftarrow M\left( a \right)$.  For each node $v\in G_{a}$, its execution function is constructed by calling $\_call\_agent(v)$, thereby defining the recursive structure.  b. **Extract inputs:** Let $d\leftarrow\sigma[A.inputs]$.  c. **Iteration subgraph execution:** For each $x$ in $d$   1. **State Injection:** $\sigma^{'}\leftarrow\sigma\cup A.idx\left[ j \right]:x_{j} \mid j\in indices\left( x \right)$. 2. **Recursive Invocation:** $o\leftarrow G_{s}.invoke\left( \sigma^{'} \right)$.   This invocation recursively triggers $\_call\_agent$ for each node in the subgraph.   3. **Output Extraction:** $v\leftarrow o[A.outputs]$ |
| 4 | **Dynamic Aggregation:** Let $R\boldsymbol{\leftarrow}\boldsymbol{\emptyset}\boldsymbol{.}$  For each output *v*: $R\leftarrow\left\{ \begin{aligned} R\oplus v,if v\in List, \\ R \vert\vert[v], otherwise \end{aligned} \right.$ |
| 5 | Return: $\Delta\sigma=A.outputs\mapsto R.$ |

**Example.** In the sentence-level NER workflow described in Section 3.2, the loop node **ner_sentence_loop** invokes the reusable subgraph **sg_ner_flair_sent** once for each sentence produced by the sentence-splitting component. Each iteration processes only the current sentence and returns sentence-level entity predictions, which are aggregated after loop completion. Similarly, , in the CID relation extraction workflow (Section 3.3), the loop node **cid_re_verify_loop** executes the subgraph **sg_cid_re_verify** independently for each candidate chemical–disease pair. Each subgraph invocation operates on isolated pair-level inputs and produces relation-level predictions that are merged into document-level outputs.

This hierarchical execution design localizes intermediate state updates, avoids premature aggregation across heterogeneous inputs, and ensures that intermediate processing steps remain modular and independently traceable throughout recursive workflow execution.

**Feature 3. Plugin integration and sandbox execution.** Local biomedical models (e.g., Flair/HunFlair2) are integrated through a unified Plugin base class whose load() method exposes named capabilities (e.g., a loaded tagger or an execution namespace) to programmatic agents and tools. Heavy-weight dependencies are executed in isolated **sandbox sidecar processes**, each running in a dedicated virtual environment (configured in meta/plugins_sandbox.json, with a default Flair sidecar exposed on port 5002). These sidecars are invoked by the main orchestrator via a restricted HTTP interface, including endpoints such as /health, /pgm/run, and /tool/run. This design ensures that PyTorch- or Flair-based execution environments are fully decoupled from the main Flask and LangGraph runtime. Lightweight programmatic agents are executed in-process under restricted PGM execution with import constraints. Plugin instances are cached after initial loading within each process to reduce overhead during repeated execution.

Custom models can be integrated either by subclassing the Plugin class in sandbox/<plugin_id>/plugins_impl.py or by extending the provided ≤50-line sandbox template and registering a new sidecar service. This design eliminates the need for Docker or external infrastructure configuration.

**Isolation guarantees.** Plugins execute in independent operating-system processes with isolated virtual environments and communicate with the main runtime exclusively through a restricted HTTP interface. As a result, dependency conflicts, memory leaks, or runtime failures occurring within a plugin are fully contained within the sandbox process and do not propagate to the main Flask/LangGraph orchestration engine. This architecture improves system robustness when multiple heterogeneous biomedical models are integrated into a single workflow.

**Feature 4 Batch experiments and reproducibility.** NeuraGraph supports batch experimentation with full execution reproducibility. Each experiment stores: the exact workflow graph executed, a snapshot of all agent and tool definitions at execution time, and per-article logs including inputs, intermediate outputs, predictions, and evaluation metrics. This snapshot mechanism ensures that subsequent re-execution uses the original experimental configuration rather than the latest version in the visual editor.

Reproducibility is further supported by versioned workflow definitions and deterministic logging of all execution artifacts. Since LLM-based components are inherently stochastic, reproducibility requires that model identity, endpoint configuration, and sampling parameters are explicitly stored and matched during re-execution.

**LLM support.** NeuraGraph supports both locally hosted models and cloud-based APIs through a unified connector abstraction. Any model accessible through a compatible HTTP endpoint can be integrated as an LLM agent without modifying workflow definitions.

**Feature 5 Evaluation metrics.** After execution, NeuraGraph automatically computes standard evaluation metrics, including precision (P), recall (R), and F1-score at both micro- and macro-average levels when ground-truth annotations are provided. These metrics are derived by comparing the $predicted (\rho)$ outputs against the $expected(\varepsilon)$ ground-truth labels, calculated as follows:

$$P=\frac{\left| \rho\cap\varepsilon\right|}{\left| \rho\right|},R=\frac{\left| \rho\cap\varepsilon\right|}{\left| \varepsilon\right|},F1=\frac{2\times P\times R}{P+R} (1)$$

where $\mid predicted\cap expected\mid$denotes the number of correct predictions (true positives), $\mid predicted\mid$ represents all items predicted, and $\mid expected\mid$represents all items in the ground truth.

Micro‑averaged metrics aggregate counts over all test instances before computing the ratios:

$$P_{micro}=\frac{\sum_{i} \mid\rho_{i}\cap\varepsilon_{i}\mid}{\sum_{i} \left| \rho_{i} \right|}, R_{micro}=\frac{\sum_{i} \mid\rho_{i}\cap\varepsilon_{i}\mid}{\sum_{i} \left| \varepsilon_{i} \right|}, F1_{micro}=\frac{2\cdot P_{micro}\cdot R_{micro}}{P_{micro}+R_{micro}} (2)$$

Macro‑averaged metrics first compute precision and recall for each instance and then take the arithmetic mean:

$$P_{macro}=\frac{1}{N}\sum_{i=1}^{N} P_{i}{, R}_{macro}=\frac{1}{N}\sum_{i=1}^{N} R_{i}, F1_{macro}=\frac{1}{N}\sum_{i=1}^{N} {F1}_{i} (3)$$

where *N* denotes the number of instances and $P_{i}$, $R_{i}$, $F1_{i}$, are the instance‑specific values obtained from Eq. (1).

Built-in micro- and macro-averaged evaluation metrics are logged throughout workflow execution. For relation extraction tasks, document-level aggregation is applied prior to metric computation to avoid pseudoreplication across sentences within the same document.

Although evaluation experiments use benchmark datasets with gold annotations, NeuraGraph also supports execution on unlabeled biomedical text. In this mode, users can load documents via parser_plain_text, execute arbitrary workflows, and obtain predicted entities, relations, summaries, or knowledge graph outputs.

For unlabeled data, all outputs are stored in experiment artifacts and reporting files, while evaluation metrics are omitted due to the absence of reference annotations.

**Feature 6 LLM-powered reporting and refinement engine.** A four-step experiment wizard (**Figure S2**) separates tuning and test evaluation and integrates batch execution, reporting, and workflow refinement.

**Figure S2.** A four-step experiment wizard.

1. **Configure.** Select a workflow, assign tuning and test splits, and validate dataset schema and gold label mappings.
2. **Baseline test and report.** Execute batch evaluation on the test split. Per-sample outputs are stored in states.json. A reporting component processes these artifacts and generates a structured Markdown baseline report, including micro/macro metrics and false positive/false negative case analysis.
3. **Tuning and optimize.** Execute the workflow on the tuning split to generate a diagnostic report. An example report is shown in **Figure S3**, including quantitative metrics, error pattern analysis (e.g., over-prediction in combination therapies), and candidate agent-level modification suggestions.

**Suggestion generation constraints.** The report prompt enforces the following constraints on modification suggestions:

- **Scope:** Only modifications applicable to existing agents, workflow graphs, or programmatic (PGM) components are allowed. External resources (e.g., UMLS, MeSH APIs, external databases) are not permitted.
- **Implementation cost:** Prefer prompt edits first, followed by lightweight programmatic checks, then workflow rewiring. Tool replacement is considered last.
- **Evidence-based:** All suggestions must be supported by per-sample error evidence from the tuning set; no hallucinated cases are allowed.
- **No shortcuts:** No use of gold labels, entity-type filters, character-position heuristics, or full-text keyword rules.
- **No hardcoding:** No reliance on specific sample IDs, entity strings, or fixed patterns.

**Refiner execution protocol.** The wizard passes the tuning report to a **refiner agent**, which generates structured modification plans. Each plan specifies: the target agent, modification type (prompt update / rule refinement), and the replacement text. The refiner is restricted to semantic modifications of LLM prompt templates and cannot modify core PGM logic or introduce heuristic filters.

Importantly, report suggestions are treated as **diagnostic signals rather than direct instructions**. The refiner may follow, combine, or override them.

**Figure S3.** Example of an LLM-powered evaluation report.

**Plan validation and testing procedure.** Each modification plan is validated through the following pipeline:

1) **Structural validation.** Plans are checked against the current agent definition. Invalid references or duplicate rules are rejected.
2) **Surgical variant execution.** The workflow is cloned, the modification is applied, and the tuning split is re-evaluated. Each variant corresponds to a single hypothesis-driven change.
3) **Acceptance criterion.** A modification is accepted only if it improves tuning macro-F1 over the baseline. Accepted changes are committed as new agent versions; rejected changes are discarded.
4) **Iteration refinement.** Accepted modifications accumulate sequentially, and later iterations build upon previous improvements. The final optimized workflow is defined by the full set of accepted modifications.

The number of evaluated plans is determined by the refiner output rather than the number of reported suggestions, ensuring empirical validation of all changes.

4. **Optimized test evaluation.** The final selected workflow is re-run on the held-out test split. The interface compares baseline and optimized precision, recall, and F1 scores and generates an optional optimized evaluation report.

## 3. Illustrative examples

To evaluate end-to-end system behavior in a realistic pharmacological text mining setting, NeuraGraph is assessed on the BioCreative V Chemical–Disease Relations (CDR) corpus under a chemical-induced disease (CID) extraction scenario from biomedical abstracts.

We define three evaluation settings:

- **Task A (NER):** named entity recognition using model predictions
- **Task B (oracle RE):** relation extraction using gold-standard entities
- **Task C (E2E):** full pipeline from predicted NER to RE

In oracle RE, gold-standard entities are used as input to the relation extraction workflow, isolating relation reasoning performance from entity recognition errors.

### 3.1. Dataset Preparation

The BioCreative V CDR corpus is used as the benchmark dataset for evaluation. It contains manually annotated chemical and disease entities and chemical–disease relations.

Dataset statistics are summarized in **Table S1**.

**Table S1.** Dataset statistics

| **Dataset** | **# Test** | **#Dev** | **Entity types** | **Relation type** |
| --- | --- | --- | --- | --- |
| CDR | 500 | 50 | Chemical, Disease | Chemical induces disease |

### 3.2. A Workflow for NER(Flair -based NER)

The named entity recognition workflow consists of three top-level components and one recursive loop subgraph (**Figure S4, Table S2**).

The workflow begins with an LLM-based sentence splitting agent, followed by a loop node that iterates over sentences and invokes a reusable subgraph executing HunFlair2-based NER. Finally, a programmatic evaluation module computes document-level entity-level precision, recall, and F1-score using merged predictions.

**Figure S4.** NER workflow structure. Agent and flow-node configuration for sentence-level entity extraction using recursive subgraph execution

**Table S2.** NER workflow components

| **Component** | **Type** | **Description** | **Inputs** | **Outputs** |
| --- | --- | --- | --- | --- |
| text_sentence_split | LLM | Split document into sentence list | article text | sentences |
| ner_sentence_loop | Loop | Iterate sentences and call subgraph | sentences | entities |
| ner_flair_sent | PGM | HunFlair2-based sentence NER | sentence | entities |
| eval_metrics | PGM | Micro/macro precision, recall, F1 | predicted / gold | metrics |

**Prompt S1** (text_sentence_split).

System: You are an English-text sentence splitter.

User template (structure):

Task: split the following biomedical abstract into complete sentences according to English grammar and punctuation.

Rules:

- Keep all content, including braces, do not remove or alter them.

- Output only a JSON array of strings, one sentence per element.

- Do not add any explanations or extra text.

- Do not add any introductory sentences like "Here is the output..."

Biomedical abstract:

{text}

**Table S3** details the source code line counts of each node in the NER task implemented by NeuraGraph.

**Table S3.** Implementation effort (NeuraGraph NER)

| **Component** | **Lines** |
| --- | --- |
| text_sentence_split | 0 |
| ner_flair_sent | 24 |
| eval_metrics | 11 |
| **Total** | **35** |

**Table S4** presents the source code line counts of the Custom Python implementation for the same NER task, with a total of 302 lines of code.

**Table S4.** Custom Python implementation (NER)

| **Component** | **Lines** |
| --- | --- |
| custom_python_ner | 63 |
| data parser | 169 |
| Data loader | 53 |
| Performance metrics calculation | 76 |
| **Total** | **361** |

### 3.3. Oracle relation extraction workflow (LLM RE)

In oracle relation extraction (oracle RE), gold-standard entities are directly provided as input to the relation extraction workflow, thereby removing errors propagated from named entity recognition (NER). This setting isolates the performance of relation verification and enables controlled comparison with end-to-end pipelines.

The oracle RE workflow consists of a structured four-stage pipeline implemented using a compound loop over candidate entity pairs **(Figure S5, Table S5):**

1. **Entity normalization and deduplication**
   Gold-standard entities are first normalized using MeSH identifiers, and duplicate or ambiguous mentions are removed while preserving canonical forms.
2. **Context-aware filtering**
   An LLM-based filtering module removes hypernyms and non-informative modifiers based on document context.
3. **Candidate pair generation**
   A programmatic module enumerates all valid chemical–disease pairs from the filtered entity set.
4. **Relation verification (loop subgraph execution)**
   Each candidate pair is processed by a reusable subgraph (sg_cid_re_verify) within a compound loop, which performs LLM-based binary relation classification and outputs structured relation tuples.

Relation-level precision, recall, and F1-score are computed from workflow-level outputs. No separate evaluation node is required, as metrics are derived directly from aggregated execution states.

**Figure S5.** Oracle RE workflow structure. Agent and flow-node specifications for the oracle relation extraction pipeline, including entity normalization, filtering, pair generation, and loop-based relation verification.

**Table S5.** Oracle RE workflow components

| **Component** | **Type** | **Description** | **Inputs** | **Outputs** |
| --- | --- | --- | --- | --- |
| e2e_entities_dedup_by_id | PGM | Normalize entities using MeSH ID and remove duplicates | entities (from CSV) | filtered_entities |
| e2e_hypernym_filter | LLM | Contextual hypernym/modifier filter (**Prompt S2**) | entities, text | filtered_entities |
| cid_pair_generate | PGM | Generate candidate chemical–disease pairs | filtered_entities | pairs |
| cid_re_verify_loop | Loop | Iterate over pairs and invoke subgraph | pairs, text | relations |
| relation_verify_llm | LLM | Binary relation classification (**Prompt S3**) | text, head, tail | label |
| relation_result_to_id_pair | PGM | Convert outputs into structured relation tuples | Result,ids | formatted relations |

**Implementation complexity. Table S6** summarizes the implementation cost of the NeuraGraph oracle RE workflow.

**Table S6.** NeuraGraph implementation cost (oracle RE)

| **Component** | **Lines** |
| --- | --- |
| e2e_entities_dedup_by_id | 20 |
| e2e_hypernym_filter | 0 |
| cid_pair_generate | 24 |
| relation_verify_llm | 0 |
| relation_result_to_id_pair | 91 |
| **Total (PGM)** | **135** |

LLM-based components are implemented as prompt configurations and therefore do not contribute to code-line counts.

**Table S7** and **Table S8** compare Custom Python and Dify tool implementations for the same task.

**Table S7.** Custom Python implementation (oracle RE)

| **Component** | **Lines** |
| --- | --- |
| custom_python_re | 68 |
| data parser | 169 |
| Data loader | 53 |
| Performance metrics calculation | 76 |
| **Total** | **366** |

Table S8 provides the source code line counts of the Dify Tool for both NER and RE tasks, serving as an additional comparison benchmark.

**Table S8.** Dify tool implementation

| **Component** | **Lines** |
| --- | --- |
| Dify_tools_re | 232 |

**Prompt S2** (e2e_hypernym_filter).

System:

You are a biomedical entity normalization expert. Do not call tools. Your reply must be ONLY the final JSON array — no reasoning, markdown, or prose.

User:

{text}

You are an expert of biomedical domain, above article is a biomedical paper.

Entities (JSON array of {"text", "id", "label"}):

{entities}

Step 1 — Drop standalone modifiers (before any pairwise comparison):

- Remove Disease rows whose text is ONLY a modifier token (temporal course, severity, stage, or similar qualifying adjective) with no disease head noun.

- Keep multi-word disease phrases even when they begin with such a modifier.

- Do not remove Chemical entities in this step.

Step 2 — Rules BEFORE pairwise hypernym removal:

1. Same id = same synonym cluster (upstream grouping). NEVER remove an entity based on comparison with another entity that has the SAME id.

2. Only compare pairs with DIFFERENT ids (same label).

3. Clinical near-synonyms or same disease event in this article: if two entities with different ids name the same condition/process, treat as '~' in BOTH orderings and KEEP both — do not remove either as a hypernym.

4. Do not remove a specific disease mention in favor of a bare modifier-only token.

Step 3 — For each pair (A,B) with the SAME label and DIFFERENT ids, substitute head and tail with the two entity texts:

1. different type → '~'

2. same type, head narrower than tail → '~'

3. same type, head broader than tail → '$'

Evaluate both (A,B) and (B,A). Remove an entity only if it receives '$' as head in comparison with another entity with a DIFFERENT id.

CRITICAL: Do NOT change or invent ids. Keep exact ids from input.

Output ONLY a JSON array:

[{"text": "...", "id": "...", "label": "Chemical"|"Disease"}]

**Prompt S3** (relation_verify_llm).

System:

You are a biomedical expert. Answer with '$' or '~' only. Do not output any other message.

User:

{text}

You are biomedical expert, refer to above biomedical article, please give answer '$' or '~' only

based on below rules. Apply all Assumptions first; if any assumption applies, answer '~' immediately and do not use Conditions.

Assumptions:

1.1 if 'patient had/exhibited {tail} on the Xth day of {head} treatment', please do not use it and find other reason.

1.2 if 'difference did not reach statistical significance compared to groups' appeared, please do not use it and find other reason.

1.3 if {head} appears only as a physiological/dietary state or experimental grouping (e.g. 'sodium depletion/repletion', 'volume depleted', 'fasted', 'control group') and not as a chemical administered to subjects, answer '~'.

1.4 if the only link between {head} and {tail} is inhibition/blockade/suppression of {head} or '{head} synthesis' by another drug (e.g. 'inhibition of {head} synthesis', 'another drug inhibits {head}'), and {tail} is attributed to that other drug or the inhibition mechanism—not to {head} itself as the causative agent—answer '~'.

1.5 if {head} is used as resuscitation, rescue therapy, antidote, or treatment for {tail} (or for local anaesthetic systemic toxicity / LAST) and {tail} is the condition being treated rather than caused by {head}, answer '~'.

Conditions:

1. if the article explicitly states that {head} caused or induced {tail} (e.g., '{head}-induced {tail}', '{head} caused {tail}', '{head} resulted in {tail}', '{head} led to {tail}'), answer '$'

2. else if the article states that the interaction between {head} and another chemical induces {tail}, answer '$'

3. else if {head} is administered together with other chemical(s) as a combination/co-treatment regimen and {tail} is reported as an adverse event, toxicity, or complication (including incidence or percentage) in patients receiving that regimen, answer '$' for {head} as a component of the regimen—even when the text attributes the event to the combination rather than {head} alone

4. else if the article mentions {tail} was reported in the percentage of patients because of {head}, and the article explicitly establishes causation (not merely listing adverse events), answer '$'

5. else if the article mentions '{head} is mediating/attenuating {tail}', answer '~'

6. else if {head} and {tail} have no relationship, answer '~'.

DO NOT output any other message.

### 3.4. End-to-End NER–RE Workflow

The sentence-level NER workflow described in Section 3.2 is connected to the CID relation extraction workflow in Section 3.3 to construct an end-to-end NER–RE pipeline (**Figure S6, Table S9**). In this setting, no gold-standard entities are used as intermediate inputs. Only raw text is provided as input, and gold annotations (entities and relations) are used exclusively for final evaluation. This design introduces realistic error propagation from NER to relation extraction, enabling end-to-end performance assessment.

The end-to-end pipeline follows a sequential execution flow:

1. **Sentence-level NER (reused subgraph).** The input text is processed using the reusable NER subgraph (sg_ner_flair_sent) described in Section 3.2, producing predicted entity mentions.
2. **Entity normalization and formatting**. Predicted label dictionaries are converted into structured entity representations for downstream processing.
3. **Synonym grouping and alias construction**. Entities are clustered into synonym groups using LLM-based similarity assignment, followed by deterministic group ID allocation. A snapshot of entity aliases is preserved before deduplication to support evaluation alignment.
4. **Entity filtering and pair generation.** Processed entities are deduplicated and filtered, then converted into candidate chemical–disease pairs.
5. **Relation verification (reused subgraph).** The CID relation verification subgraph (sg_cid_re_verify, Section 3.3) is applied to each candidate pair.
6. **Evaluation**. Predicted relations are normalized using the stored alias mapping and compared against gold-standard relations to compute precision, recall, and F1-score.

**Figure S6.** End-to-end NER–RE workflow. Integrated pipeline combining sentence-level NER, entity normalization, synonym grouping, pair generation, and relation verification under a unified execution graph.

**Table S9.** End-to-end workflow components

| **Component** | **Type** | **Description** | **Inputs** | **Outputs** |
| --- | --- | --- | --- | --- |
| sg_ner_flair_sent | Subgraph | Sentence-level NER (see Table S2) | text, labels | entities (label dict) |
| ner_entities_to_re_format | PGM | Convert Flair label dict to entity list format [{text, label, id}] | entities | entities (list) |
| e2e_entities_passthrough | PGM | Initial deduplication of NER outputs | entities | filtered_entities |
| e2e_synonym_filter | LLM | Assign synonym clusters (**Prompt S4**) | entities, text | filtered_entities |
| e2e_entities_assign_group_ids | PGM | Assign deterministic group IDs | entities, filtered_entities | filtered_entities |
| e2e_entity_aliases_snapshot | PGM | Store alias mapping for evaluation | filtered_entities | alias_map |
| sg_cid_re_verify | Subgraph | Relation verification (**Table S5**) | filtered_entities, text, … | relations |
| eval_metrics_relation | PGM | Compute micro/macro metrics using gold_relations | predictions, ground truth | metrics |

**Prompt S4** (e2e_synonym_filter).

System:

You are a biomedical entity normalization expert. Do not call tools. Your reply must be ONLY the final JSON array — no reasoning, markdown, or prose.

User:

Text:

{text}

Entities (JSON array of {"text", "id", "label"}):

{entities}

Task: assign each input entity to a synonym CLUSTER. Grouping only — do NOT drop any entity.

A cluster = entities that would map to the same MeSH concept in BC5CDR (same chemical, or same disease), based on this article.

Same label + same cluster id when:

- Abbreviation, generic name, or trade name of the same drug/substance.

- Morphological variant of the same drug (e.g. -related, -associated, -induced).

- Clinical near-synonyms for the SAME disease event or pathological process in context: e.g. organ injury, organ toxicity, organ inflammation, organ damage, or named syndrome wording that refers to one outcome.

- One drug-adverse-event narrative in the article → one Disease cluster id for all Disease mentions describing that same reaction (do not split injury vs toxicity vs inflammation vs named disease if they refer to one event).

- Multi-word disease phrase and its head term → same cluster.

- Bare temporal/severity/course modifier alone → same cluster id as the disease phrase it modifies in the text, not a separate cluster.

Different cluster ids only when the article clearly denotes distinct chemicals, or distinct unrelated disease conditions/events.

Output — ONE row per INPUT entity (count must match input):

- text = exact input surface form (preserve casing).

- id = cluster key shared by all cluster members (stable canonical phrase from the group; no MeSH/CHEBI/DOID codes).

- label = Chemical or Disease.

CRITICAL:

- Every input entity exactly once; do NOT omit entities.

- All members of one cluster use the identical id string.

- Do not invent ontology codes.

Output ONLY a JSON array:

[{"text": "...", "id": "...", "label": "Chemical"|"Disease"}]

### 3.5. Full performance evaluation (pharmacovigilance use case)

#### 3.5.1. Use case

Given PubMed-style title–abstract input, the system performs:

1. Named entity recognition (Chemical, Disease)
2. Candidate chemical–disease relation extraction
3. Evaluation with micro/macro precision, recall, and F1 at article level

This setting models a pharmacovigilance scenario for extracting adverse drug reactions from biomedical literature.

#### 3.5.2. Evaluation setup

We evaluate NeuraGraph on the BioCreative V CDR corpus using three experimental settings:

1. **NER (Task A).** We evaluate Flair-based NER (Section 3.2) and an LLM-only NER baseline on the 500-test set. Performance is measured using entity-level micro/macro precision, recall, and F1-score.
2. **CID–RE (Task B).** We evaluate the LLM-based relation extraction workflow (Section 3.3) under an oracle setting using gold-standard entities, reporting both baseline and tuning-guided optimized variants derived from the development set.
3. **End-to-end CID–RE (Task C).** We construct a full pipeline by chaining Task A NER outputs into the optimized CID–RE workflow from Task B, and compare against an external baseline using the PubTator3 API for both NER and RE.

The full experiment configuration is summarized in **Table S10**.

**Table S10.** Experiment registry (BC5CDR test, n=500)

| **Task** | **Method** | **Runner / script** | **Dataset** | **Experiment id** |
| --- | --- | --- | --- | --- |
| A | Flair NER | wf_doc_ner_flair_sent_eval | test-500 | 064f47ac-*** |
| A | LLM NER^*1^ | wf_doc_ner_llm_eval | test-500 | aba808f2-*** |
| B | LLM RE (baseline) | wf_cid_re_llm_linear | test-500 | 678a855f-*** |
| B | LLM RE(tuning baseline) | wf_cid_re_llm_linear | dev-50 | opt_base_tune_* |
| B | LLM RE  (refinement candidates) | wf_cid_re_llm_linear | dev-50 | opt_cand_*** |
| B | LLM RE (optimized) | wf_cid_re_llm_linear_opt_* | test-500 | opt_best_*** |
| C | E2E (Flair NER to optimized RE) | wf_e2e_flair_opt_re | test-500 | bffea244-*** |
| C | E2E (PubTator 3) ^*2^ | wf_e2e_pubtator_re | test-500 | 5c3bad58-*** |

**Note: ^*1^** LLM NER replaces sentence-level Flair inference with a single document-level LLM call for baseline comparison. ^*2^ PubTator3-based pipeline integrates external NER and RE components under the same evaluation interface.

#### 3.5.3. Results

**Task A** **(NER).** Two NER workflows were evaluated on the 500-test set (**Table S11**). The Flair-based pipeline achieves **0.779 micro/macro F1**, significantly outperforming the LLM-only baseline (0.656 / 0.667), demonstrating the advantage of domain-specific biomedical sequence labeling models for entity recognition.

**Table S11.** NER performance (test, n=500)

| **Method** | **Micro-P** | **Micro-R** | **Micro-F1** | **Macro-P** | **Macro-R** | **Macro-F1** |
| --- | --- | --- | --- | --- | --- | --- |
| Flair NER | **0.725** | **0.840** | **0.779** | **0.737** | **0.844** | **0.779** |
| LLM NER | 0.640 | 0.673 | 0.656 | 0.672 | 0.690 | 0.667 |

**Task B (CID–RE, oracle setting).** Pharmacovigilance CID–RE was tuned using a stratified 50-article subset of the development split via the experiment wizard (Feature 6). The optimization process follows a report-guided refinement pipeline that separates diagnostic analysis from model updating. A baseline CID–RE workflow was first executed on the test split (n=500), and a tuning baseline was generated on the development subset (n=50). The LLM-powered reporting engine then produced a structured diagnostic report **(Figure S3**), summarizing error patterns and proposing four candidate modifications:

**S1 (physiological substances & lab markers):** Targeting **relation_verify_llm**, proposing exclusion of endogenous biomarkers (e.g., creatinine, serotonin) unless explicitly administered as drugs.

**S2 (treatment/rescue drug detection):** Extending Assumption 1.5 to include sedatives and supportive care agents (e.g., diazepam, morphine, naloxone), enforcing negative classification when the head entity is used therapeutically.

**S3 (negation boundary in PGM logic):** Strengthening negation detection in **relation_result_to_id_pair** by requiring proximity constraints on both head and tail entities.

**S4 (combination therapy effects):** Clarifying that drug–drug combinations associated with adverse events should still be classified as positive CID relations.

The refiner component processes the report and converts selected suggestions into executable modification plans, restricted to LLM prompt-level edits only. Under these constraints, S1 and S3 were filtered out (S1 introduces new assumptions; S3 targets programmatic logic). S2 and S4 were retained as candidate plans.

Tuning results on the development split (Table S12) show:

- Plan 1 (S2) decreased macro-F1 to 0.605 (Δ −0.036) and was rejected.
- Plan 2 (S4) improved macro-F1 to 0.666 (Δ +0.024) and was accepted.

The final accepted modification was applied to **relation_verify_llm**, refining Condition 2 to better capture reversed-order drug–drug interaction patterns (e.g., “cisapride–diltiazem interaction”).

**Table S12.** Tuning performance (dev, n=50)

| **Stage** | **Experiment id** | **Macro-F1** | **ΔMacro-F1** |
| --- | --- | --- | --- |
| Tuning baseline | opt_base_tune_81781d50 | 0.642 | — |
| Plan 1 (S2) | opt_cand_toxidrome_15_420244af | 0.605 | −0.036 |
| Plan 2 (S4) | opt_cand_ddi_c2_8cb2b66e | 0.666 | +0.024 |

**Optimized Test:** The accepted modification was frozen into **relation_verify_llm**, and the optimized workflow was re-evaluated on the test set (n=500). Results in **Table S13** show consistent improvements over the baseline across both micro- and macro-averaged metrics.

**Table S13.** CID–RE performance on test set (oracle entities, n=500)

| **Method** | **Micro-P** | **Micro-R** | **Micro-F1** | **Macro-P** | **Macro-R** | **Macro-F1** |
| --- | --- | --- | --- | --- | --- | --- |
| LLM RE (baseline) | 0.583 | 0.750 | 0.656 | 0.610 | 0.757 | 0.642 |
| LLM RE (optimized) | **0.603** | **0.761** | **0.673** | **0.626** | **0.791** | **0.666** |

**Task C** **(End-to-end CID–RE).** The end-to-end CID–RE pipeline chains Flair-based NER outputs from Task A into the optimized CID–RE workflow from Task B. Unlike Task B, no gold-standard entities are provided; only raw text and gold relations are used for evaluation, such that NER errors propagate into downstream pair generation and relation verification.

A PubTator3-based pipeline (**wf_e2e_pubtator_re**) is used as an external baseline under the same evaluation protocol. This enables comparison between NeuraGraph and an established biomedical annotation service in an end-to-end setting.

NeuraGraph achieves a micro-F1 of 0.635 and macro-F1 of 0.663, substantially outperforming PubTator3 (micro-F1: 0.394, macro-F1: 0.422), demonstrating the effectiveness of the proposed hybrid NER–RE pipeline.

The detailed results are reported in **Table S14**.

**Table S14.** End-to-end CID–RE performance (test, n=500)

| **Method** | **Micro-P** | **Micro-R** | **Micro-F1** | **Macro-P** | **Macro-R** | **Macro-F1** |
| --- | --- | --- | --- | --- | --- | --- |
| E2E (Flair NER to optimized RE) | **0.543** | **0.765** | **0.635** | 0.632 | 0.781 | **0.663** |
| E2E (PubTator3) | 0.281 | 0.660 | 0.394 | 0.344 | 0.699 | 0.422 |

## 4. Pre-built component inventory

To support low-code workflow construction, NeuraGraph provides a library of reusable components, including pre-built agents, reusable subgraphs, reference workflows, callable tools, and native dataset parsers. These components enable rapid assembly of end-to-end biomedical text-mining pipelines with minimal custom programming.

The distribution includes **67** pre-built agents (**36** LLM-based and **31** programmatic/PGM; see **Tables S15-S16**)), **10** reusable subgraphs (**Table S17**) orchestrated via native loop and branch flow nodes, **23** reference workflows (**Table S18**), **12** callable tools (**Table S19**). Native parsers for CDR, ChemDisGene, and plain text (**Table S20**).

### 4.1. Agents

**Table S15.** LLM agents (36)

| **No.** | **ID** | **Name** | **Description** |
| --- | --- | --- | --- |
| 1 | agent_refiner | Agent Refiner (LLM) | Generate actionable agent modifications from experiment diagnostics. |
| 2 | chemdisgene_re_chem_disease_affects | ChemDisGene RE chem_disease:affects | Verify ChemDisGene chem_disease:affects pairs; returns numeric answer code. |
| 3 | chemdisgene_re_chem_gene_activity | ChemDisGene RE chem_gene:activity | Verify ChemDisGene chem_gene:activity pairs; returns numeric answer code. |
| 4 | chemdisgene_re_chem_gene_aff_bind | ChemDisGene RE chem_gene:affects^binding | Verify ChemDisGene chem_gene:affects^binding pairs; returns numeric answer code. |
| 5 | chemdisgene_re_chem_gene_aff_expr | ChemDisGene RE chem_gene:affects^expression | Verify ChemDisGene chem_gene:affects^expression pairs; returns numeric answer code. |
| 6 | chemdisgene_re_chem_gene_aff_local | ChemDisGene RE chem_gene:affects^localization | Verify ChemDisGene chem_gene:affects^localization pairs; returns numeric answer code. |
| 7 | chemdisgene_re_chem_gene_dec_metab | ChemDisGene RE chem_gene:decreases^metabolic_processing | Verify ChemDisGene chem_gene:decreases^metabolic_processing pairs; returns numeric answer code. |
| 8 | chemdisgene_re_chem_gene_expression | ChemDisGene RE chem_gene:expression | Verify ChemDisGene chem_gene:expression pairs; returns numeric answer code. |
| 9 | chemdisgene_re_chem_gene_inc_activity | ChemDisGene RE chem_gene:increases^activity | Verify ChemDisGene chem_gene:increases^activity pairs; returns numeric answer code. |
| 10 | chemdisgene_re_chem_gene_inc_metab | ChemDisGene RE chem_gene:increases^metabolic_processing | Verify ChemDisGene chem_gene:increases^metabolic_processing pairs; returns numeric answer code. |
| 11 | chemdisgene_re_chem_gene_transport | ChemDisGene RE chem_gene:transport | Verify ChemDisGene chem_gene:transport pairs; returns numeric answer code. |
| 12 | chemdisgene_re_gene_disease_marker | ChemDisGene RE gene_disease:marker/mechanism | Verify ChemDisGene gene_disease:marker/mechanism pairs; returns numeric answer code. |
| 13 | chemdisgene_re_gene_disease_therapeutic | ChemDisGene RE gene_disease:therapeutic | Verify ChemDisGene gene_disease:therapeutic pairs; returns numeric answer code. |
| 14 | chemdisgene_re_hypernyms | ChemDisGene RE hypernyms | Verify same-type hypernym relation between head/tail; answer $ or ~. |
| 15 | chemdisgene_re_synonym | ChemDisGene RE synonym | Verify same-type synonym/alias relation between head/tail; answer $ or ~. |
| 16 | e2e_hypernym_filter | E2E Hypernym Filter (LLM) | Remove hypernyms and standalone modifiers from entity list using article context; keep only the most specific entities per concept. |
| 17 | e2e_synonym_filter | E2E Synonym Filter (LLM) | Assign every input entity to a synonym cluster (shared id); do not drop entities. |
| 18 | kg_triple_extract_llm | Triple Extraction (LLM) | Biomedical knowledge-graph triple extraction |
| 19 | ner_chemdisgene_llm | NER (ChemDisGene) | ChemDisGene NER: type/entity per line |
| 20 | ner_comparison_report | NER Comparison Report | Generates a comparison report between Flair and LLM NER results |
| 21 | ner_from_tree_llm | NER from Dependency Tree (LLM) | Extract entities from CoNLL-U dependency tree |
| 22 | ner_llm | NER (LLM, configurable types) | Extract biomedical named entities from text |
| 23 | ontology_entity_link | Entity Linking / Normalization (LLM) | Canonicalize entity mentions for knowledge-graph linking |
| 24 | ontology_hypernym_filter | Hypernym Filter (LLM) | Identify same-type hypernyms by MeSH id; drop hypernym rows from entity list |
| 25 | ontology_synonym_resolve | Synonym Resolution (LLM) | Biomedical abstract synonym extraction (aligned with synonym_extraction) |
| 26 | relation_extract_llm | Relation Extraction (LLM) | Strict biomedical CID relation verifier for chemical–disease pairs |
| 27 | relation_from_tree_llm | Relation Extraction from Tree (LLM) | Extract [head entity, verb, tail entity] triples from CoNLL-U dependency tree |
| 28 | relation_verify_llm | Relation Verification (LLM) | CID induce verifier (llmre prompt_templates.json RE.induce, lines 17-23) |
| 29 | report_comparator | Report Comparator (LLM) | Compare baseline vs candidate experiment outcomes and report quality. |
| 30 | report_experiment | Experiment Report (LLM) | Generate a structured experiment diagnosis report from full workflow artifacts. |
| 31 | report_experiment_tool | Experiment Report (LLM + Tools) | Generate a structured experiment diagnosis report from full workflow artifacts. |
| 32 | syntax_dep_parse | Dependency Parse (CoNLL-U) | English sentence to CoNLL-U dependency tree |
| 33 | text_coreference | Coreference Resolution (LLM) | Biomedical abstract coreference resolution |
| 34 | text_sentence_split | Sentence Split (LLM) | English sentence splitting for biomedical abstract |
| 35 | text_summarize | Text Summarization (LLM) | Summarize text into 2-3 sentences |
| 36 | text_word_segment | Word Segmentation (LLM) | English word segmentation |

**Table S16.** PGM agents (31)

| **No.** | **ID** | **Name** | **Description** |
| --- | --- | --- | --- |
| 1 | chemdisgene_answer_map | ChemDisGene Answer Map (PGM) | ChemDisGene Answer Map (PGM) |
| 2 | chemdisgene_ner_parse | ChemDisGene NER Parse (PGM) | ChemDisGene NER Parse (PGM) |
| 3 | chemdisgene_pair_generate | ChemDisGene Pair Generator (PGM) | ChemDisGene Pair Generator (PGM) |
| 4 | chemdisgene_result_to_relation | ChemDisGene Result to Relation (PGM) | ChemDisGene Result to Relation (PGM) |
| 5 | cid_entities_dedupe_mesh | CID Entities Dedupe (MeSH id) | CID Entities Dedupe (MeSH id) |
| 6 | cid_pair_generate | CID Pair Generator (PGM) | CID Pair Generator (PGM) |
| 7 | dataset_chemdisgene_build | ChemDisGene Dataset Builder (PGM) | ChemDisGene Dataset Builder (PGM) |
| 8 | dataset_cid_tuning_build | CID Tuning Dataset Builder (PGM) | CID Tuning Dataset Builder (PGM) |
| 9 | e2e_entities_assign_group_ids | E2E Assign Synonym Group IDs (PGM) | E2E Assign Synonym Group IDs (PGM) |
| 10 | e2e_entities_dedup_by_id | E2E Entities Dedup By ID | Deduplicate entities by shared id; keep shortest surface form per id group. |
| 11 | e2e_entities_passthrough | E2E Entities Passthrough | Dedupe Flair NER entities and use surface text as id when MeSH id is missing (E2E RE path). |
| 12 | e2e_entity_aliases_snapshot | E2E Entity Aliases Snapshot (PGM) | Preserve all synonym surface forms + shared numeric ids for metrics lookup (before dedup). |
| 13 | eval_flair | Auto FLAIR NER Metrics | Counts unique FLAIR NER entities per label and returns total and per-label entity counts. |
| 14 | eval_llm | Auto LLM NER Metrics | Counts unique LLM NER entities per label and returns total and per-label entity counts. |
| 15 | eval_metrics | Evaluation Metrics (PGM) | Computes NER precision, recall, and F1 by comparing predicted and expected entity sets via MetricsCalculation. |
| 16 | eval_metrics_relation | Relation Set Metrics (PGM) | Evaluates chemical–induced-disease relation sets by normalizing and comparing predicted relations to ground truth. |
| 17 | eval_metrics_segment | Segmentation Metrics (PGM) | Scores pipe-delimited word segmentation by boundary precision, recall, F1, and segmentation error analysis. |
| 18 | eval_pair_generate | Evaluation Pair Generator (PGM) | Generates all chemical–disease evaluation pairs from expected Chemical and Disease entity lists. |
| 19 | kg_rdf_export | RDF-JSON Export (PGM) | Exports knowledge-graph triples to RDF-JSON with node labels and subject–predicate–object edges. |
| 20 | kg_triple_merge | Triple Merge (PGM) | Merges pipe-delimited relations with entity-link canonicalization and deduplicates head–predicate–tail triples. |
| 21 | kg_triple_persist | Triple CSV Persistence (PGM) | Persists merged knowledge-graph triples to CSV under result with head, verb, and tail columns. |
| 22 | llm_link_bulk_update | Bulk LLM Link Update (PGM) | Bulk-updates LLM connector links on selected agents from a source model to a target, optionally dry-run. |
| 23 | merge_metrics | Merge Flair and LLM Metrics | Combines FLAIR and LLM NER metric dictionaries into a single merged_metrics report object. |
| 24 | ner_entities_to_re_format | NER Entities to RE Format | Converts Flair NER label dict {Chemical:[...], Disease:[...]} to the entity list format [{text, label, id}] expected by the CID RE pipeline. |
| 25 | ner_flair_aggregate | Flair NER Aggregator | Aggregates per-sentence FLAIR NER outputs into document-level label-to-unique-entity-text dictionaries. |
| 26 | ner_flair_doc | NER (Flair, document) | Runs HunFlair2 NER on a document split into sentences and returns deduplicated entities per label. |
| 27 | ner_flair_sent | NER (Flair, sentence) | Tags one sentence with HunFlair2 NER and returns predicted entity texts grouped by label. |
| 28 | relation_extract_pubtator | Relation Extraction (PubTator) | Extracts chemical–disease relations from text or PMID using PubTator3 and local entity annotations. |
| 29 | relation_result_to_id_pair | Relation Result -> ID Pair | Emits head_id/tail_id CID relation lines when verification is positive, with negation and weak-association guards. |
| 30 | relation_verify_to_pair | Relation Verify → Entity Pair | Maps a positive relation verification to canonical head and tail entity IDs from entity_link. |
| 31 | report_format_json | Result Formatter (PGM) | Formats original text and summary into JSON with character lengths for reporting pipelines. |

### 4.2 Reusable subgraphs

**Table S17.** Reusable subgraphs (10)

| **No.** | **ID** | **Name** | **Description** |
| --- | --- | --- | --- |
| 1 | sg_cid_re_verify | CID RE verify pair | Verify one Chemical–Disease pair; output MeSH id pair when verdict is induces. |
| 2 | sg_e2e_cid_re | E2E CID RE (subgraph) | Subgraph: passthrough → synonym → assign ids → alias snapshot → dedup → hypernym → pair gen → verify loop. |
| 3 | sg_e2e_flair_ner | E2E Flair NER (subgraph) | Subgraph: sentence split → loop HunFlair2 per sentence → RE format entities. Input: text + labels. Output: entities as [{text, label, id}]. |
| 4 | sg_e2e_pubtator_re | E2E PubTator3 RE (subgraph) | Subgraph: PubTator3 API → CID relations. Input: text + pmid. Output: relations as head_id / CID / tail_id lines. |
| 5 | sg_ner_flair_sent | Sentence Flair NER | Run ner_flair_sent on one sentence (loop body). |
| 6 | sg_preprocess_inner | Inner preprocess loop body | Format / pass-through inner loop step. |
| 7 | sg_re_preprocess | RE preprocess + NER | Nested inner loop then ner_llm (outer loop body for doc RE). |
| 8 | sg_re_tree | Tree-based RE | syntax_dep_parse → relation_from_tree_llm per sentence. |
| 9 | sg_relation_verify | Relation verify pair | relation_verify_llm → relation_verify_to_pair for one head/tail pair. |
| 10 | sg_chemdisgene_re_verify | ChemDisGene RE verify pair | Branch on rel_template to ChemDisGene RE verify agents; map answer to relation line. |

### 4.3. Reference workflows

**Table S18.** Reference workflows (23)

| **No.** | **ID** | **Name** | **Description** |
| --- | --- | --- | --- |
| 1 | wf_chemdisgene_ner_llm_eval | ChemDisGene NER Eval (LLM) | ChemDisGene NER with type/entity prompt and entity dict metrics. |
| 2 | wf_chemdisgene_re_llm_linear | ChemDisGene RE Pipeline (linear) | Gold entities → pair generation → foreach RE verify (template branch) → relation metrics. |
| 3 | wf_cid_ner_llm_eval | CID NER Eval (LLM) | Chemical/Disease NER with LLM and generic metrics. |
| 4 | wf_cid_re_branch | CID RE (multi-branch gate) | NER then 4-way branch gate before relation extraction. |
| 5 | wf_cid_re_llm_linear | CID RE Pipeline (linear) | Gold entities → e2e dedup by id → e2e hypernym filter (with text context) → pair list → foreach RE verify → id pairs. Upgraded from wf_e2e_flair_opt_re agents. |
| 6 | wf_cid_re_llm_linear_opt_20260529_143039_r2 | CID RE Pipeline (linear) [optimized] | Gold entities → e2e dedup by id → e2e hypernym filter (with text context) → pair list → foreach RE verify → id pairs. Upgraded from wf_e2e_flair_opt_re agents. |
| 7 | wf_cid_re_llm_linear_opt_20260604 | CID RE Pipeline (linear) [optimized v2] | Gold entities → hypernym filter → pair list → foreach RE verify → id pairs. Optimized on 50-stratified dev articles. relation_verify_llm prompt: reject endogenous substances, accept AE reporting language. |
| 8 | wf_doc_ner_flair_eval | Doc NER Eval (Flair) | Document Flair NER with metrics (replaces bio_ner_graph). |
| 9 | wf_doc_ner_flair_sent_eval | Doc NER Eval (Flair, sentence) | Sentence split → loop HunFlair2 per sentence → entity metrics (Table S2 / Figure S2). |
| 10 | wf_doc_ner_llm_eval | Doc NER Eval (LLM) | Split document then LLM NER with metrics. |
| 11 | wf_doc_ner_loop_branch | Doc NER loop + branch | Sentence split → loop Flair NER → 4-way branch → optional LLM → metrics. |
| 12 | wf_doc_re_nested_branch | Doc RE (nested loop + branch) | Outer loop with nested preprocess + NER, 4-way branch, then RE. |
| 13 | wf_e2e_flair_opt_re | E2E Flair NER + Optimized RE | Flair sentence-split NER → optimized CID RE (v0008 prompt) → relation metrics. For Table S18. |
| 14 | wf_e2e_pubtator_re | E2E PubTator3 RE | Compose sg_e2e_pubtator_re (PubTator3 API → relations) → relation metrics vs gold_relations. For Table S18 comparison. |
| 15 | wf_flair_vs_llm_ner | Flair vs LLM NER Comparison | Sentence split, Flair loop NER, LLM NER, per-path metrics, and ner_comparison_report on the same document. |
| 16 | wf_general_report_linear | Summarize + format | Summarize text and pack into JSON result. |
| 17 | wf_kg_flair_full | KG Build (Flair NER) | Flair doc NER → entity link → triple extract → merge → RDF. |
| 18 | wf_kg_llm_full | KG Build (LLM NER) | LLM NER → entity link → RE → triple merge → RDF export. |
| 19 | wf_kg_syntax_loop | KG from syntax (sentence loop) | Coreference → split → loop tree-RE subgraph → CSV persist. |
| 20 | wf_re_pubtator_dev10 | PubTator RE Dev (10) | BC5CDR dev.txt (10 articles): gold entities → PubTator3 relation extraction → relation pair metrics vs gold_relations. |
| 21 | wf_re_pubtator_eval | PubTator RE Eval | Gold entities → PubTator relation extraction → relation pair metrics. |
| 22 | wf_re_verify_llm_loop | RE Verify (pair loop) | Generate evaluation pairs → loop verify subgraph → metrics. |
| 23 | wf_word_seg_llm_eval | Word Segmentation Eval | LLM word segmentation with boundary metrics. |

### 4.4 Callable tools

**Table S19.** Callable tools (12).

| **No.** | **ID** | **Name** | **Description** |
| --- | --- | --- | --- |
| 1 | agent_change_impact_trace | Agent Change Impact Trace | Estimate per-agent metric contribution from version_map and two experiment states. |
| 2 | agent_version_guard | Agent Version Guard | Policy-based guard for keep/alert/rollback decisions using metric deltas. |
| 3 | dataset_cid_tuning_build | CID Tuning Dataset Builder | Build a stratified CID tuning CSV (and optional test-remain set) from PubTator gold dev.txt. |
| 4 | dataset_sampler_stratified | Dataset Sampler Stratified | Stratified sample by text length, entity density, and relation density for quick A/B validation. |
| 5 | error_case_exporter | Error Case Exporter | Export worst-k error cases from states into markdown and jsonl payloads for review/regression. |
| 6 | fn_fp_bucket_analyzer | FN FP Bucket Analyzer | Bucket FN/FP into boundary/type/relation/missed-recall style categories with examples. |
| 7 | merge_heads_tails_to_entities | Merge Heads & Tails to Entities | Merge two lists of head/tail entities into a unified 'text,type,mesh' string format, one entity per line. |
| 8 | metrics_delta_compare | Metrics Delta Compare | Compare baseline and candidate states, return precision/recall/f1 deltas and significantly degraded samples. |
| 9 | prompt_patch_apply_safe | Prompt Patch Apply Safe | Apply structured prompt patch (append/replace/remove-rules) with validation and safety checks. |
| 10 | report_quality_scorer | Report Quality Scorer | Score report quality on evidence, executability, and traceability. |
| 11 | tool_ner_flair | NER by Flair | Input a sentence string, and label string like "Chemical,Disease", the tool will do NER task and return a dict like {"Chemical":["a","b"], "Disease":["c","d"] } |
| 12 | tool_pubtator_relation_extract | PubTator Relation Extract | Call NCBI PubTator3 API (<https://www.ncbi.nlm.nih.gov/research/pubtator3-api>) to extract chemical–disease relations from a PubMed PMID or text. Returns relations as head / CID / tail lines. |

### 4.5 Native dataset parsers

**Table S20.** Native dataset parsers (3).

| **No.** | **ID** | **Name** | **Description** |
| --- | --- | --- | --- |
| 1 | parser_cdr | CDR (PubTator) | Load BioCreative V CDR PubTator .txt articles with gold entities and CID relations (CIDParser). |
| 2 | parser_chemdisgene | ChemDisGene | Load ChemDisGene article .txt files with companion .tsv annotation trees (ChemDisGeneParser). |
| 3 | parser_plain_text | Plain text (raw upload) | Load user-uploaded plain-text .txt from data/raw for batch runs without bundled gold files. |
